# Assessment of Physician's Knowledge of Potential Drug-Drug Interactions: An Online Survey in China

**DOI:** 10.3389/fmed.2021.650369

**Published:** 2021-03-01

**Authors:** Jing Yuan, Chunying Shen, Chengnan Wang, Gang Shen, Bing Han

**Affiliations:** ^1^Minhang Hospital & Department of Clinical Pharmacy at School of Pharmacy, Fudan University, Shanghai, China; ^2^Department of Neurosurgery, Minhang District Central Hospital, Shanghai, China

**Keywords:** drug-drug interaction, PDDI, adverse drug event, survey, knowledge, medication

## Abstract

**Background:** Drug interactions are the most common preventable cause of adverse drug reaction, which may result in drug toxicity or undesired therapeutic effect with harmful outcomes to patients. Given the rising use of combination therapies, the main objectives of this study were to estimate the degree to which physicians can identify potential drug-drug interactions (PDDIs) correctly and to describe the common source of information used by physicians when they need to check PDDIs.

**Methods:** A cross-sectional survey utilizing a self-administered online questionnaire was conducted among physicians in China. Participants were asked to classify 20 drug pairs as “no interaction,” “may be used together with monitoring,” “contraindication,” and “not sure.” We also collected data on the physician's source of information and altitude toward the PDDIs. An ordinary least square regression model was performed to investigate the potential predictors of PDDI knowledge.

**Results:** Eligible questionnaires were obtained from 618 physicians. The respondents classified correctly 6.7 out of 20 drug pairs, or 33.4% of the drug interactions investigated. The number of drug pairs recognized by respondents was ranged from 0 to 16. The percentage of physicians who recognized specific drug pairs ranged from 8.3% for no interactions between conjugated estrogens and raloxifene, to 64.0% for the interaction between dopamine and phenytoin. When the respondents want to check PDDI information, the most commonly used source of information was package inserts (*n* = 572, 92.6%), followed by the Internet or mobile Apps (*n* = 424, 68.6%), consultation with clinical pharmacists (*n* = 384, 62.1%), medical textbooks (*n* = 374, 60.5%), knowledge base in Chinese (*n* = 283, 45.8%), and other physicians (*n* = 366, 59.2%). In the multiple regression analysis, the significant predictors of a higher number of recognized drug pairs were years of practice and altitudes toward PDDIs.

**Conclusion:** In this online survey accessing physician's ability to detect PDDIs, less than half of the drug pairs were recognized, indicating unsatisfactory level of knowledge about the clinically significant drug interactions. Continuing education and accessible electronic database can help physicians detecting PDDIs and improve drug safety.

## Introduction

A drug-drug interaction occurs when the pharmacokinetic or pharmacodynamic properties of a drug are altered when two or more drugs are taken simultaneously (1, 2). The interactions between two or more drugs may exist antagonistic or synergistic effect, both of which lead to drug toxicity or undesired therapeutic effect with harmful outcomes to patients (3–5). The potential drug-drug interactions (PDDIs) were identified in around 30% of the prescriptions in the outpatient (6) and oncology departments in China (6, 7). Even though the prevalence data is largely lacking in China, the PDDIs may be more prevalent in intensive care settings (8) and hematology (9, 10), based on the data from other countries. Patients experienced a PDDI are generally associated with a longer hospital stay (11) and higher medical costs (12, 13), leading to substantial financial burden on healthcare systems as well as on patients and society (14).

Physicians play a key role in preventing and reducing the risk of PDDIs and associated adverse outcomes. Unlike other countries such as the United States (U.S.), the clinical decision support system, which assists clinicians to detect PDDIs, is not commonly available in China. Physicians rely on their own knowledge to recognize PDDIs when writing a prescription. Even with the help of clinical decision support system, the performance of such system in terms of accurately identifying PDDIs was still unknown in China. Unsatisfactory performance of these systems has been reported in U.S (15, 16). For example, large variations in the electronic databases were reported (17–20), resulting in confusions among clinicians. In a recent study comparing three commercial knowledge databases – First DataBank (FDB), Micromedex, and Multum, it was found that the overlap of drug pairs in all these three knowledge bases was as low as 5%, and the number of alerts generated for serious PDDIs were ranged from 25 to 145 alerts per 1,000 prescriptions (18). Despite the suboptimal performance of computerized systems, clinicians tend to override the automated alerts and may ignore PDDIs (21).

To our best knowledge, however, no studies have attempted to test physician's knowledge of PDDIs in China. Internationally, few studies assessed the ability of clinicians to detect PDDIs and their sources of drug information (22–25). In a postal survey of U.S. prescribers, response from a 16-item questionnaire suggested that prescribers were able to detect 42.7% of drug combinations (22). In another survey using simulated prescription profiles, 67% of 2-drug combinations were categorized correctly by pharmacists (23). In addition to the PDDI knowledge, the source of information contributes to the irrational use of medicines. For example, if physicians rely on the drug information from pharmaceutical companies rather than evidence-based guidelines, incorrect medication use may occur (26). But limited data is available for where the physicians obtained their PDDI information in China.

China has become the second largest pharmaceutical market in the world. With the rising use of medications, it is becoming increasingly urgent to navigate potential ways reducing the risk of PDDIs. In 2019, the National Health Commission (NHC) released opinions on strengthening rational use of medicines to address the emerging issue in patient safety. As such, understanding the physician's knowledge level of PDDIs is warranted to develop evidence-based strategies and policies to improve patient care. Therefore, the objective of this study was 3-fold: [1] to estimate the degree to which physicians can identify PDDIs correctly; [2] to describe the source of information that physicians used; [3] to understand their altitude with respect to improving knowledge level of PDDIs.

## Materials and Methods

### Study Design

This study employed a cross-sectional study design. We carried out an anonymous, online survey that was open to practicing physicians in Shanghai, China, from November 1 to December 15, 2020. This study was approved by the Institutional Review Boards of Minhang Hospital of Fudan University. The study protocol and gave an exemption from full review. Written informed consent was waived for this study because of the anonymous survey approach. This study followed the Checklist for Reporting Results of Internet E-Surveys (CHERRIES) (27).

### Survey Questionnaire

The questionnaire, which accessed the knowledge level of PDDIs, was created based on previous studies and clinical practices in China (22, 24, 28). This questionnaire has been reviewed by an expert panel that consisted of physician, pharmacists, outcomes researcher. The questionnaire included three sections; the first section was to ask physician's characteristics, including age, gender, education, years of practice, and specialty. The second section was to access physician's knowledge for PDDIs. Participants were asked to classify the 20-drug pairs into four categories: “no interaction” (*n* = 5), “have interaction, may be used together but with monitoring” (*n* = 8), “contraindication” (*n* = 7), and “not sure.” Each correct answer was given one point, with a maximum score of 20. These DDI pairs are commonly used in the literature testing the knowledge level of PDDIs (22, 24, 28). We selected these drug pairs because they are frequently used in China with significant clinical impacts. The last section was to understand the source of information. As various sources of drug information may be used to check for PDDIs, the participants were expected to choose multiple answers from the options given. We also evaluated physician's altitude on the prevention of PDDIs. The 5-point Likert scale was used to allow the participants to express the extent they agree or disagree with the four statements regarding their altitude toward of avoiding PDDIs in practice. The questionnaire was shown in the Appendix.

### Participants Recruitment

To reach a representative sample of physicians, following the previous research (29), we first selected 20 clinical pharmacists as the original delivers who invited the physicians in their units to participate. Physicians working in both community clinics and hospitals were invited to participate. Then we sent out private messages via WeChat that included a link to web-based questionnaire through an internet survey portal (https://www.wjx.cn/). WeChat is the largest social media platform in China and has been widely used to distribute online surveys (30, 31). Each WeChat account was allowed to answer the questionnaire once to avoid multiple response from the same participants. A total of 900 participants were invited in this survey and 709 physicians participated the survey. The response rate was 78.8%.

The returned questionnaire was considered as eligible if [1] all the questions related to the PDDIs were answered; [2] the time spent on answering the questionnaire was within the range of 2–20 min, which was considered as typical timestamps. In the questionnaire development stage, we collected the feedbacks from a small group of physicians on whether the survey questions were clear. The typical timestamps were determined by the time they spent on completing the questionnaire. This approach has been applied in previous studies (30, 32). If the respondents submitted the same answers for all the PDDIs questions, then the questionnaire was excluded from the analysis.

### Data Analysis

For descriptive analysis, frequency distributions (e.g., percentage) was used to describe categorical variables and means were used to describe continuous variables, respectively. Fisher's exact test was for categorical variables and Student's *t*-test was used for continuous variables. The mean was calculated to describe the score of PDDI questions. We also constructed an ordinary least square regression model to examine the potential predictors of PDDI knowledge, including physician's demographics, specialty, type of hospitals, practices, and their altitude toward PDDIs. The selection of these factors was based on the practices in China. The number of drug pairs categorized correctly was used as dependent variable, and the physician's self-reported characteristics and altitude on the PDDIs were included as independent variables. On the basis of parsimony, the interaction terms were not included in the regression model because the interacting effect of independent variables is difficult to interpret (22). Statistical significance was determined at a-level of 0.05. All statistical analyses were performed using SAS 9.4.

## Results

### Characteristics of Respondents

After discarding 88 questionnaires that deemed ineligible based on predefined criteria, 618 (or 88%) of 706 questionnaires were included in the analysis. As shown in Table 1, the majority (42.6%) of respondents were aged between 30 and 39 years old; 353 (57.1%) were female, and 256 (41.4%) held a graduate degree. Most of physicians worked in community hospitals (38.8%) and tertiary hospitals (38.8%), and 25.9. 18.8, 35.1, and 14.6% of the respondents having practiced for <5 years, 5–9 years, 10–19 years, and more than 20 years, respectively. 40.0% of the respondents were internal medicine or family medicine physicians.

**Table 1:** Self-reported characteristics of physicians who participated in the survey (*n* = 618).

**Characteristics**	**Number of participants (*n*)**	**Percentage (%)**
**AGE**		
20–29	127	20.6
30–39	263	42.6
40–19	171	27.7
50+	57	9.2
**GENDER**		
Male	265	42.9
Female	353	57.1
**EDUCATION**		
High school	1	0.2
College/Bachelor degree	361	58.4
Graduate/Master degree	256	41.4
**TYPE OF HOSPITAL**		
Community hospital	240	38.8
Secondary hospital	66	10.7
Tertiary hospital	240	38.8
Private hospital/others	72	11.7
**YEARS OF PRACTICE**		
<5	160	25.9
5–9	116	18.8
10–19	217	35.1
20–30	90	14.6
>30	35	5.7
**SPECIALTY**		
Internal/general medicine	247	40.0
Surgery	92	14.9
Emergency medicine	44	7.1
Others	235	38.0

### Knowledge of PDDIs

Table 2 presents the frequencies (percentages) of respondents choosing each answer of the 20-drug pairs. On average, the respondents classified correctly 6.7 out of 20 pairs, or 33.4% of the drug interactions investigated. The number of drug pairs recognized by respondents was ranged from zero to 16. The percentage of physicians who recognized specific drug pairs ranged from 8.3% for no interactions between conjugated estrogens and raloxifene, to 64.0% for the interaction between dopamine and phenytoin.

**Table 2:** Frequencies (percentages) of physician's response to PDDIs^a^.

**Drug pairs**	**No interaction**	**May be used together but with monitoring**	**Contraindication**	**Not sure**
Acetaminophen/codeine and amoxicillin	**124 (20.1%)**	337 (54.5%)	28 (4.5%)	129 (20.9%)
Warfarin and sulfamethoxazole/trimethoprim	44 (7.1%)	**376 (60.8%)**	104 (16.8%)	94 (15.2%)
Warfarin and digoxin	**79 (12.8%)**	377 (61.0%)	77 (12.5%)	85 (13.8%)
Digoxin and amiodarone	26 (4.2%)	**334 (54.0%)**	185 (29.9%)	73 (11.8%)
Cyclosporine and rifampicin^*^	48 (7.8%)	381 (61.6%)	**108 (17.5%)**	82 (13.2%)
Digoxin and itraconazole	56 (9.1%)	**390 (63.1%)**	94 (15.2%)	78 (12.6%)
Digoxin and sildenafil	**53 (8.6%)**	371 (60.0%)	114 (18.5%)	81 (13.0%)
Simvastatin and itraconazole	62 (10.0%)	361 (58.3%)	**113 (18.3%)**	83 (13.3%)
Sildenafil and isosorbide mononitrate^*^	47 (7.6%)	373 (60.3%)	**102 (16.5%)**	97 (15.6%)
Conjugated estrogens and raloxifene	**51 (8.3%)**	387 (62.6%)	79 (12.8%)	101 (16.3%)
Theophylline and ciprofloxacin^*^	83 (13.4%)	**357 (57.8%)**	75 (12.1%)	103 (16.7%)
Pimozide and ketoconazole^*^	70 (11.3%)	366 (59.1%)	**73 (11.8%)**	110 (17.7%)
Warfarin and fluconazole	59 (9.6%)	**384 (62.1%)**	82 (13.3%)	93 (15.0%)
Alprazolam and itraconazole^*^	87 (14.1%)	373 (60.4%)	**65 (10.5%)**	93 (15.0%)
Digoxin and clarithromycin^*^	73 (11.8%)	**382 (61.7%)**	52 (8.4%)	112 (18.0%)
Warfarin and sulfinpyrazone^*^	50 (8.1%)	**383 (61.9%)**	101 (16.3%)	85 (13.7%)
Dopamine and phenytoin	40 (6.5%)	**396 (64.0%)**	95 (15.4%)	88 (14.2%)
Fexofenadine and metoprolol	**58 (9.4%)**	396 (64.0%)	62 (10.0%)	103 (16.6%)
Itraconazole and quinidine	40 (6.5%)	382 (61.7%)	**100 (16.2%)**	97 (15.6%)

a *Bold text indicates the correct answers based on the Lexi-Interact*.

* *Indicates that these drug pairs were considered as clinically significant*.

For the five drug combinations without interactions, 9.8% of the respondents answered correctly. For the 15 drug combinations that were considered as having interactions, which include both “contraindication” and “have interaction but may be used together with monitoring,” 41.2% of them were categorized correctly by the respondents. Of particular, 15.1% of respondents categorized correctly for the six drug pairs having contradicted interactions, while 60.2% of the respondents categorized them as “needs to have close monitoring.” For the seven drug pairs that were deemed with clinical significance by the expert panel (33), the majority of respondents detected the interactions, but 10% of the respondents still categorized as “no interaction” and 15% of them answered “not sure.”

### Source of PDDI Information

**Figure 1:**
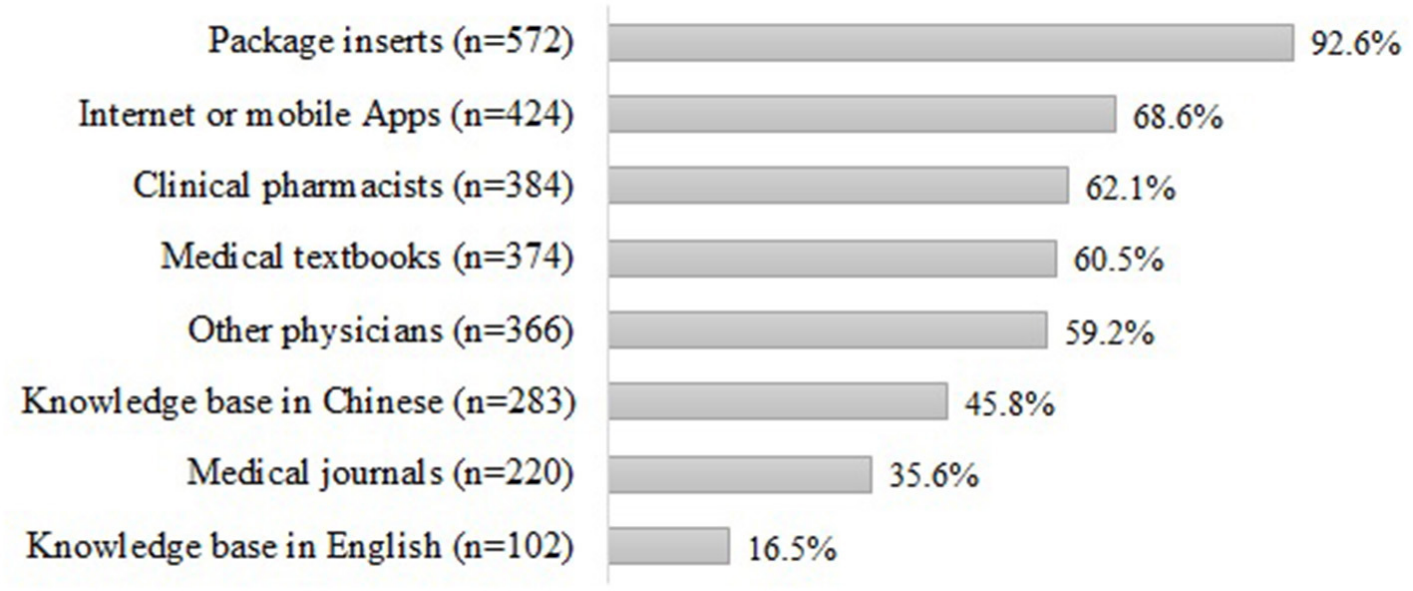
Source of information for PDDIs^a^. ^a^Sum may not be 100% because respondents were allowed to choose multiple answers.

The respondents' source of PDDI information was shown in Figure 1. When the respondents wanted to check PDDI information, the most commonly used source of information was package inserts (*n* = 572, 92.6%). The less commonly used information sources were Internet or mobile Apps (*n* = 424, 68.6%), consultation with clinical pharmacists (*n* = 384, 62.1%), medical textbooks (*n* = 374, 60.5%), knowledge base in Chinese (*n* = 283, 45.8%), and consultation with other physicians (*n* = 366, 59.2%).

### Altitude Toward PDDIs

**Figure 2:**
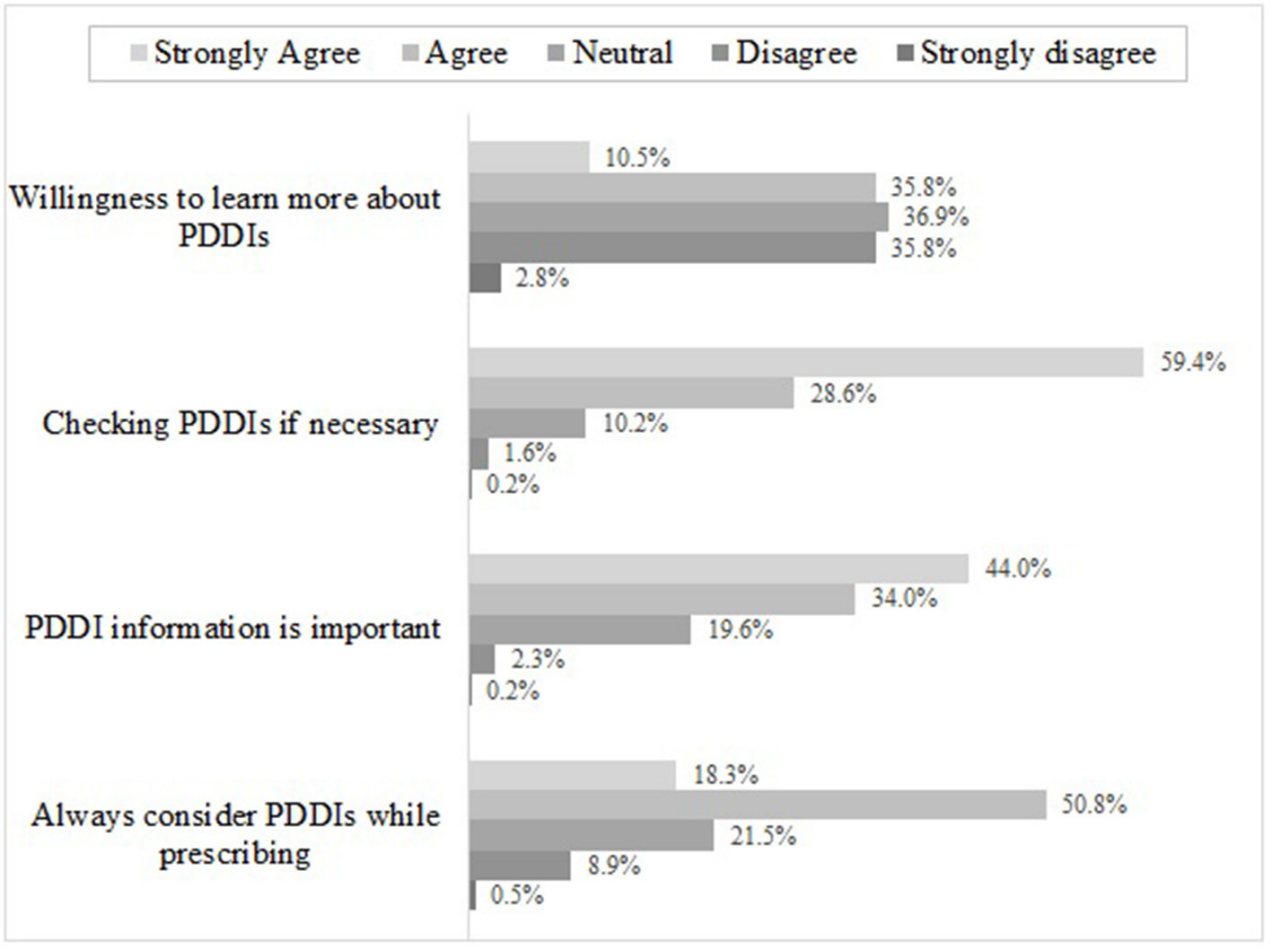
Physician's altitude toward PDDIs^*^. ^*^Sum may not be 100% because respondents were allowed to choose multiple answers.

As shown in Figure 2, 88.0% of respondents reported that they always checked PDDIs while prescribing for patients, among them, 28.6% agreed with the statements and 59.4% strongly agreed, respectively. 69.1% of respondents agreed that they would consider PDDIs while prescribing. Less than half of the respondents agreed that the PDDI information were useful for their practice, but 78.0% of them had the willingness to improve their knowledge for PDDIs.

### Predictors of PDDI Knowledge

In the multiple regression analysis, it revealed that significant predictors of a higher number of recognized drug pairs were years of practice (*p* = 0.015) and altitudes toward PDDIs (Table 3). Respondents who reported to check references for PDDIs detected more drug interactions than those who did not look for PDDIs (*p* = 0.029). Respondents who reported to consider PDDIs while prescribing for patients had lower score for PDDIs than those who did not (*p* = 0.011).

**Table 3:** Predictors of the knowledge level for PDDIs^a^.

**Characteristics**	**Estimate**	**Standard Error**	***p***
**AGE**			
20–29 years	Ref	–	–
30–39 years	−0.04	0.25	0.875
40–19 years	−0.53	0.33	0.111
50+ years	1.44	0.51	0.005^*^
**GENDER**			
Male	Ref	–	–
Female	0.14	0.16	0.368
**EDUCATION**			
High school	−2.96	1.84	0.108
College/Bachelor degree	0.22	0.18	0.242
Graduate/Master degree	Ref	–	–
**YEARS OF PRACTICE**			
<5 years	Ref	–	–
5–9 years	−0.29	0.25	0.261
10–19 years	0.12	0.27	0.670
20–30 years	0.56	0.38	0.138
>30 years	1.40	0.57	0.015^*^
**TYPE OF HOSPITAL**			
Community hospital	Ref	–	–
Secondary hospital	0.55	0.26	0.035^*^
Tertiary hospital	0.18	0.21	0.389
Private hospital/others	−0.28	0.27	0.298
**SPECIALTY**			
Internal/general medicine	Ref	–	–
Surgery	−0.33	0.24	0.162
Emergency medicine	−0.36	0.31	0.245
Others	−0.15	0.17	0.397
**ALTITUDE**			
Always consider PDDIs while prescribing	−0.24	0.10	0.011^*^
Willingness to learn PDDIs	0.12	0.08	0.147
Considering PDDI as useful	−0.14	0.11	0.200
Looking for PDDI information	0.26	0.12	0.029^*^

a *Predictors were estimated from the an ordinary least square regression*.

* *p < 0.05*.

## Discussion

In this online survey accessing physician's ability to recognize PDDIs, less than half of the interacting drug combinations were recognized by the physicians who responded to the survey, which is consistent with previous studies conducted in the U.S. (22) and Central Saudi Arabia (28). Based on the participants' response, it seems that some potentially harmful drug interactions may not be detected by many physicians. For the seven drug combinations that are considered as contradicted, nearly 80% of the respondents categorized incorrectly. This insufficient detection of contradicted drug combinations could be partly explained by that the use of these drugs requires close monitoring, and hence a large proportion (around 60%) of respondents chose “use with monitoring” rather than “contradicted.” However, up to 25% of the physicians still remained unsure or unaware of these serious PDDIs. In addition, the participants were asked to restrict the use of references, which may contribute the relatively poor performance of recognizing PDDIs (22, 34).

Our findings also revealed that package inserts were the most commonly used information source for physicians when they need to check PDDIs, which is a major risk factor for incorrect medication use (26). As the electric databases or computerized systems are not widely available in China, it would be difficult for physicians to stay current with the best available evidence. With the busy working schedule, it appears to be impossible for physicians to check package inserts for every potential drug interaction they found, leading to substantial threats to the patient safety. Therefore, easily accessed scientific resources should be available for physicians to improve drug safety. Even with the assistance of the computerized systems to detect PDDIs, preventing DDIs is still difficult especially when more drugs are a part of a patient's daily schedule (35). Pharmacists and physicians tend to be desensitized to the alerts that are given by the software systems (36), mainly because they are given the option to override those alerts with mild or insignificant clinical outcomes (35, 36). Overriding alerts may become a habit for many pharmacists; in some clinical settings, the override rates could be as high as 71.9%, which may rise the tendency of a pharmacist or physician to override a harmful PDDI (37). Hence, the burning issue of drug interaction won't be easily fixed by the implementation of the computerized system, a multi-facetted approach is necessary to improve drug safety.

This study also attempted to investigate the predictors of physician's PDDI knowledge. Due to small sample size, few predictors, including years of practice, type of hospital, and altitude toward PDDIs, were statistically significant. Higher scores for PDDIs were reported among physicians working in the secondary hospitals, potentially because they tend to provide care to a wide spectrum of non-surgical conditions and might be more familiar with the PDDIs included in the questionnaire. Noticeably, our analyses indicated the strong association between self-reported willingness to check references and knowledge of PDDIs as demonstrated by correct responses to drug pairs. Our finding highlighted the importance of raising awareness of PDDIs among physicians, possibly through continuing education with a specific focus on the most harmful PDDIs. Furthermore, two-thirds of physicians would consult with pharmacists if they have question regarding PDDIs. Recently, the pharmacist's role in improving patient care has been recognized in China. For example, the review of prescription by clinical pharmacists was mandated in tertiary hospitals in Shanghai. The physician-pharmacist collaboration model, in which pharmacists are included in the multidisciplinary heath care team, has been piloted in some tertiary hospitals. Most of PDDIs are preventable through quick response and effective team collaborations, therefore, more efforts should be pioneered in building up the physician-pharmacist collaboration to improve pharmaceutical care (7).

There are several limitations in this analysis. First, our findings have limited generalizability and may not reflect the general doctor population in terms of their level of knowledge about PDDIs. Despite of the efforts in the distribution of questionnaire, only a small proportion of physicians in China responded. Hence, the findings cannot be generalizable to all Chinese physicians. Second, selection bias may exist because physicians who were familiar with PDDIs were more likely to participate the survey. As such, it will be impossible to understand the knowledge level of those physicians who did not participate the survey. Third, the questionnaire used to test the knowledge level of PDDIs is not validated, even though it was developed based on the existing studies (22, 24, 28). The 20-drug pairs might not be adequate to reflect the extent of knowledge applicable to the huge number of PDDIs. Hence, the physician's knowledge about PDDIs may not be well-accessed in this study. However, these DDI pairs have significant clinical impacts and every practicing physicians should be familiar with them. Last, we only included a couple of predictors for PDDI knowledge level, the lacking of confounders may cause inaccurate results of the multivariate regression model.

In conclusion, this study indicates unsatisfactory knowledge level about clinically significant drug interactions among physicians. This study also provides insights into where the physicians obtain their drug information. Continuing education and accessible electronic database can help physicians detecting PDDIs and improve drug safety.

## Data Availability Statement

The raw data supporting the conclusions of this article will be made available by the authors, without undue reservation.

## Ethics Statement

The studies involving human participants were reviewed and approved by The Institutional Review Boards of Minhang Hospital of Fudan University. Written informed consent for participation was not required for this study in accordance with the national legislation and the institutional requirements.

## Author Contributions

JY and CS: concept and design. CS, BH, CW, and GS: acquisition, analysis, or interpretation of data. CS and JY: drafting of the manuscript. JY, GS, BH, CW, and CS: critical revision of the manuscript for important intellectual content. JY: statistical analysis. JY and BH: administrative, technical, or material support and supervision. All authors approved the final manuscript as submitted and agree to be accountable for all aspects of the work.

## Conflict of Interest

The authors declare that the research was conducted in the absence of any commercial or financial relationships that could be construed as a potential conflict of interest.
