# Supplementary material for: Assessment of Physician's Knowledge of Potential Drug-Drug Interactions: An Online Survey in China
Source: Front Med (Lausanne). 2021 Mar 1;8:650369. doi: 10.3389/fmed.2021.650369 (PMC7957001; doi:10.3389/fmed.2021.650369)
Supplement: Supplementary file 1 [file Table_1.docx]

Appendix. Survey questionnaire

1. **Please indicate your age**

- 20-29 years
- 30-39 years
- 40-19 years
- 50+ years

1. **Please indicate your gender**

- Male
- Female

1. **Please indicate your highest education**

- High school
- College/Bachelor degree
- Graduate/Master degree

1. **How many years have you practiced?**

- <5 years
- 5-9 years
- 10-19 years
- 20-30 years
- >30 years

1. **Please indicate the type of hospital you are working:**

- Community hospital
- Secondary hospital
- Tertiary hospital
- Private hospital/others

1. **What is your specialty**

- Internal/general medicine
- Surgery
- Emergency medicine
- Others

1. **In the past three months, which of the following sources of information have you used** **when you need to check drug-drug interactions?**

- Knowledge base in English
- Medical journals
- Knowledge base in Chinese
- Other physicians
- Medical textbooks
- Clinical pharmacists
- Internet or mobile Apps
- Package inserts

1. **Please indicate the extent to which you agree or disagree with the following statement**

|  | Strongly Agree | Agree | Neutral | Disagree | Strongly Disagree |
| --- | --- | --- | --- | --- | --- |
| I always consider PDDIs while prescribing |  |  |  |  |  |
| I think PDDI information is important for my practice |  |  |  |  |  |
| I always check PDDIs when I am not sure about it |  |  |  |  |  |
| I am willingness to learn more about PDDIs |  |  |  |  |  |

1. **Please select the best option to describe the drug-drug interactions below:**

|  | No interaction | With monitoring | Contraindicated | Not sure |
| --- | --- | --- | --- | --- |
| Acetaminophen/codeine and Amoxicillin |  |  |  |  |
| Warfarin and Sulfamethoxazole/trimethoprim |  |  |  |  |
| Warfarin and Digoxin |  |  |  |  |
| Digoxin and Amiodarone |  |  |  |  |
| Cyclosporine and rifampicin |  |  |  |  |
| Digoxin and Itraconazole |  |  |  |  |
| Digoxin and Sildenafil |  |  |  |  |
| Simvastatin and Itraconazole |  |  |  |  |
| Sildenafil and isosorbide mononitrate |  |  |  |  |
| Conjugated estrogens and raloxifene |  |  |  |  |
| Theophylline and ciprofloxacin |  |  |  |  |
| Pimozide and ketoconazole |  |  |  |  |
| Warfarin and Fluconazole |  |  |  |  |
| Alprazolam and itraconazole |  |  |  |  |
| Digoxin and Clarithromycin |  |  |  |  |
| warfarin and Sulfinpyrazone |  |  |  |  |
| Dopamine and phenytoin |  |  |  |  |
| Fexofenadine HCL and metoprolol |  |  |  |  |
| Itraconazole and quinidine |  |  |  |  |
